# Agreement in cardiovascular risk rating based on anthropometric parameters

**DOI:** 10.1590/S1679-45082015AO3349

**Published:** 2015

**Authors:** Endilly Maria da Silva Dantas, Cristiane Jordânia Pinto, Rodrigo Pegado de Abreu Freitas, Anna Cecília Queiroz de Medeiros

**Affiliations:** 1 Universidade Federal do Rio Grande do Norte, Natal, RN, Brazil.

**Keywords:** Anthropometry, Body weights and measures, Cardiovascular diseases, Risk assessment, Sex

## Abstract

**Objective:**

To investigate the agreement in evaluation of risk of developing cardiovascular diseases based on anthropometric parameters in young adults.

**Methods:**

The study included 406 students, measuring weight, height, and waist and neck circumferences. Waist-to-height ratio and the conicity index. The kappa coefficient was used to assess agreement in risk classification for cardiovascular diseases. The positive and negative specific agreement values were calculated as well. The Pearson chi-square (χ^2^) test was used to assess associations between categorical variables (p<0.05).

**Results:**

The majority of the parameters assessed (44%) showed slight (k=0.21 to 0.40) and/or poor agreement (k<0.20), with low values of negative specific agreement. The best agreement was observed between waist circumference and waist-to-height ratio both for the general population (k=0.88) and between sexes (k=0.93 to 0.86). There was a significant association (p<0.001) between the risk of cardiovascular diseases and females when using waist circumference and conicity index, and with males when using neck circumference. This resulted in a wide variation in the prevalence of cardiovascular disease risk (5.5%-36.5%), depending on the parameter and the sex that was assessed.

**Conclusion:**

The results indicate variability in agreement in assessing risk for cardiovascular diseases, based on anthropometric parameters, and which also seems to be influenced by sex. Further studies in the Brazilian population are required to better understand this issue.

## INTRODUCTION

As from the 1960s’, cardiovascular diseases (CVD) have represented a primary cause of death in Brazil, with a progressive increase in the number of cases. In 2013, approximately 300 thousand Brazilian individuals died due to CVD, and the Southeast and Northeast regions, respectively, ranked first and second.^1^

Considering this situation that is prevalent all over the world, some efforts have been made to develop and identify techniques and markers that can be used to evaluate cardiovascular risk, thus allowing triage of the population to initiate follow-up as early as possible.^2,3^

The World Health Organization also suggests the use of anthropometric measurements for surveillance of risk factors of chronic disease, such as CVD, besides recommending to begin monitoring as early as possible, particularly considering the increased prevalence of obesity and chronic diseases among younger people.^4,5^

Aiming to put this recommendation into practice, various anthropometric parameters have been proposed and studied in an effort to better evaluate central obesity and the risk for CVD, such as waist circumference (WC),^6,4^ neck circumference (NC),^7,8^ conicity index (CI),^9^ and waist-to-height ratio (WHR).^10,11^

Despite promising results, however, most of the studies on the theme are carried out on populations that are middle-aged or older, and there are scarce investigations on young adults or that evaluate agreement and applicability of the cutoff points and parameters that exist in the younger population.^3,7,10,11^

## OBJECTIVE

To investigate the agreement in evaluating risk of developing cardiovascular diseases based on different anthropometric parameters in young adults.

## METHODS

This is an exploratory, quantitative and cross-sectional study that evaluated 406 students (135 males and 271 females) of the *Universidade Federal do Rio Grande doNorte* (UFRN).

The research protocol was approved by the Humans Research Ethics Committee of the UFRN, under official opinion number 122,536 and CAAE: 06531412.4.0000.5537, and all volunteers signed the Informed Consent Form.

The inclusion criteria for participation in the study were aged 18 years or more, regularly enrolled in an undergraduate course at the university, and not present with any limitation that would hinder the collection of anthropometric measurements. Volunteer recruitment was done by announcement in classrooms and in lounges of the university.

The anthropometric assessment was made by trained evaluators who checked body weight and height, NC and WC. All measurements were taken in duplicate to obtain the mean. In case of disagreement between the values obtained, a third measurement was taken, and the divergent value was excluded in the calculation of the mean value.

To verify height, a stadiometer (Sanny^®^, São Paulo, Brazil) supported on an anodized aluminum rod was used, with a tripod support and measurement capacity of 115 to 210cm. Volunteers were placed with their backs to the rod, adopting the Frankfurt plane.^12^

Weight was measured on portable digital scales (Plenna^®^, São Paulo, Brazil), with a capacity for 150kg. The students were instructed to remove shoes and any additional objects.^12^

Body circumferences were measured using a non-elastic anthropometric tape made of fiberglass, with a latch and length of 200cm. The WC was verified at the midpoint between the iliac crest and the last rib, with the reading done at the end of expiration.^13^In order to check NC, the participants were positioned standing on the Frankfurt plane with their arms loose along the sides of their bodies, and the measurement was made above the thyroid cartilage prominence.^14^

The formulas used to calculate the CI and WHR, as well as the cutoff values used to assess risk of development CVD, based on anthropometric parameters, are described on chart 1.

**Chart 1:** Anthropometric markers and reference values for cardiovascular risk assessment

Anthropometric markers	Formula	Reference value for risk	Reference
WC (cm)	-	≥94 for men	ABESO^(13)^
		≥80 for women	
NC (cm)	-	≥39.6 for men	Stabe et al.^(7)^
		≥36.1 for women	
WHR	WHR=WC(cm)/H(cm)^(12)^	≥0.52 for men	Pitanga^(15)^
		≥0.53 for women	
CI	CI=WC(m)/0,109√ W(kg)/H(m)	1.25 for men	Pitanga^(15)^
		1.18 for women	

WC: waist circumference; NC: neck circumference; WHR: waist-to-height ratio; H: height; CI: conicity index; W: weight.

The statistical analysis was conducted using the software Statistical Package for the Social Science (SPSS), version 19.0. The results were expressed as mean, standard deviation, median, percentiles, and percentages.

The evaluation of agreement in classification of cardiovascular risk - based on different anthropometric parameters, was performed by calculating the Kappa coefficient, considered the best index for this type of evaluation.^16^To interpret this measurement, the following criteria proposed by Altman^17^ were adopted, which classify the Kappa coefficient as five categories, according to strength of agreement: very good (0.81 to 1.00); good (0.61 to 0.80); moderate (0.41 to 0.60); fair (0.21 to 0.40), and poor (<0.20).

As per recommended by Feinstein,^16^ in order to better contextualize the Kappa value, the observed agreement and the positive (PA) and negative (NA) specific agreement values were also calculated. The PA and NA values are used to help identify the possible sources of disagreement between the results obtained with Kappa statistics and the general agreement value.^16,18^

In order to investigate possible associations among the categorical variables, the Pearson’s chi-squared χ^2^ test was used. Statistical significance was considered when the p value was <0.05.

## RESULTS

Most of the study population (66.75%) was composed of women, with a mean age of 21.1±3.22 years. Table 1 shows the anthropometric profile of the participants.

**Table 1:** Characterization of the population by sex

Parameter	Men (n=135)		Women (n=271)	
	Mean±SD	Median (p25-p75)	Mean±SD	Median (p25-p75)
Age (years)	20.8±2.85	20.00 (19.00-22.00)	21.1±3.22	20.00 (19.00-22.00)
BMI (kg/m^2^)	24.40±3.70	24.19 (21.89-26.49)	22.99±3.72	22.49 (20.51-25.01)
WC (cm)	81.70±9.23	81.00 (75.00-87.40)	76.29±9.12	75.00 (70.00-82.00)
NC (cm)	36.95±3.29	37.00 (35.00-39.00)	32.02±2.36	31.90 (30.50-33.00)
WHR	0.47±0.05	0.47 (0.44-0.50)	0.47±0.05	0.47 (0.43-0.51)
CI	1.15±0.06	1.16 (1.11-1.20)	1.15±0.07	1.14 (1.10-1.19)

The results were expressed as mean, standard deviation, median, 25^th^ percentile, and 75^th^ percentile. SD: standard deviation; p25: 25^th^ percentile; p75: 75^th^ percentile; WC: waist circumference; NC: neck circumference; WHR: waist-to-height ratio; CI: conicity index.

Table 2 shows the results of the agreement in classification of risk of developing CVD, based on the different anthropometric parameters evaluated. According to the Kappa coefficient classification, no case was found of very good agreement among the parameters evaluated. The best agreement (good) was between the parameters WC and WHR, both for the general population and between sexes.

**Table 2:** Agreement in classification for risk of developing cardiovascular disease, based on anthropometric measurements

Parameters		Observed agreement	Kappa coefficient (95%CI)	PA	NA
WC *versus* NC	Total	0.78	0.25 (0.14-0.35)	0.35	0.87
	Male	0.89	0.52 (0.32-0.72)	0.62	0.94
	Female	0.72	0.16 (0.07-0.26)	0.24	0.83
WC *versus* CI	Total	0.85	0.60 (0.51-0.69)	0.70	0.90
	Male	0.92	0.52 (0.28-0.76)	0.56	0.96
	Female	0.81	0.58 (0.48-0.68)	0.72	0.86
WC *versus* WHR	Total	0.88	0.66 (0.57-0.75)	0.73	0.93
	Male	0.93	0.72 (0.56-0.88)	0.76	0.96
	Female	0.86	0.64 (0.54-0.74)	0.72	0.91
WHR *versus* NC	Total	0.84	0.35 (0.23-0.48)	0.43	0.91
	Male	0.84	0.46 (0.26-0.66)	0.55	0.91
	Female	0.85	0.28 (0.14-0.43)	0.34	0.91
WHR *versus* CI	Total	0.80	0.43 (0.33-0.53)	0.55	0.87
	Male	0.86	0.37 (0.16-0.58)	0.42	0.92
	Female	0.77	0.45 (0.34-0.55)	0.58	0.84
NC *versus* CI	Total	0.73	0.12 (0.02-0.21)	0.24	0.84
	Male	0.85	0.27 (0.05-0.49)	0.33	0.92
	Female	0.67	0.13 (0.05-0.21)	0.21	0.79

General: n=406; male: n=135; female: n=271. 95%CI: 95% confidence interval; PA: positive specific agreement value; NA: negative specific agreement value; WC: waist circumference; NC: neck circumference; CI: conicity index; WHR: weight-to-height ratio.

Most of the parameters evaluated (44%) presented with Kappa coefficient values classified as fair and/or poor agreement, and low values of NA.

**Figure 1:**
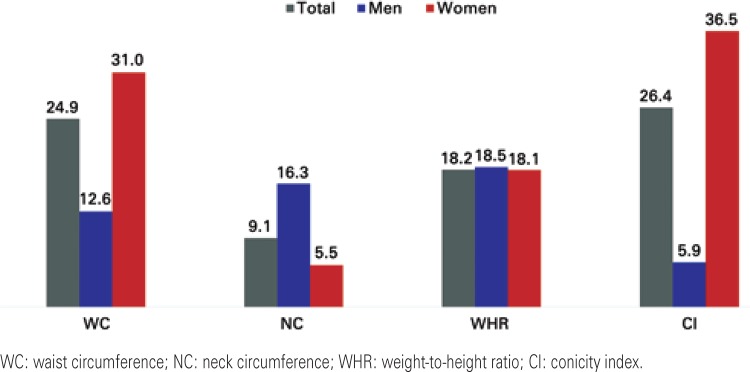
Prevalence of increased risk of developing the disease

As to prevalence of increased risk of CVD, as presented on figure 1, a great variability was observed in risk, depending on sex and the anthropometric parameter used in evaluation. The greatest classification of risk for the general population and male sex was observed in the CI assessment, whereas for females, it was in the WC. The greatest discrepancy found for men and women and the general population was between WC and NC (25.5%).

The results of the χ^2^test showed a significant association between risk of developing CVD and the female sex, when using the parameters WC (χ^2^(1)=16.33; p<0.001) and CI (χ^2^(1)=43.48; p<0.001); and between the risk of developing CVD and the male sex, when evaluating the parameter NC (χ^2^(1)=12.60; p<0.001). No association was found between the risk for CVD and sex, according to the WHR (χ^2^(1)=0.01; p>0.05).

## DISCUSSION

In our study, despite a high proportion of agreement observed in the risk classification for CVD, a strong tendency towards Kappa coefficient values classified as fair and/or poor was noted.

This situation was called the “Kappa paradox”, and the evaluation of other parameters is recommended, such as the PA and NA values, in order to identify possible sources of disagreement in the Kappa statistics. Therefore, based on the assessment of PA and NA values, it is possible to visualize consistency among observers and/or methods, especially regarding decision-making in opposite directions. That is, the degree to which they agree with the classification of who is positive (when a given factor is presented), as well as when they agree as to the classification of one who does not have this factor.^16,18^

In the case, for example, of assessing the risk classification for CVD based on WC and NC parameters, a high degree of agreement was found, with low Kappa coefficient values. Nevertheless, the evaluation of PA and NA values allowed the identification that this discrepancy probably was due to the low values of the PA, indicating low agreement in the evaluation among the methods of those who were classified as having increased risk for CVD.

This was a tendency that crossed all the discrepancies found (low Kappa values with high observed agreement), in which low values of PA were noted in the comparison among the methods (WC *versus* CI; WHR *versus* NC; WHR *versus* CI; NC *versus* CI). Thus, despite the high agreement values, when observing the Kappa statistics result and the PA and NA values, a low agreement was noted in the risk classification for CVD among the methods.

This variability in agreement in assessing risk of CVD also seems related to the combination parameter/sex evaluated. There was an association between the female sex and risk of CVD when the evaluation was made using the parameters of CI (p<0.001) and WC (p<0.001), and association with the male sex, when evaluating the NC (p<0.001). This finding was observed together with an ample variation in the prevalence of risk for CVD, estimated by different methods.

Although these results show a tendency towards low agreement in assessing risk of CVD between the association of parameters evaluated, some Brazilian studies have demonstrated the existence of an association among these measurements and the risk for CVD. A cross-sectional study, with 968 undergraduate students, in the State of Maranhão, found a correlation between WC and WHR and cardiovascular risk factors, such as high triglycerides and smoking.^19^ Another study that included 155 adults aged between 20 and 60 years, residing in the State of Rio Grande do Sul, verified the association between NC and risk factors for CVD, as well as increased values of WC.^20^ Other national projects also reported a positive correlation between anthropometric parameters that are predictors of central obesity and factors related to increased risk of CVD, such as high blood pressure and increased blood lipids.^21-23^

From this view, a large prospective study carried out in the United States with 49,032 men and women aged under 61 years, found that a greater quantity of body fat conferred a greater risk for CVD, both in men and in women, regardless of the parameter chosen for the evaluation.^24^

Nonetheless, according to the results of the present study, depending on which anthropometric parameter is used to evaluate central obesity, and depending on the sex of the individual evaluated, there may be large differences in the result of risk assessment for CVD.

Despite there being a consensus about validity of using anthropometric measurements and their positive impact as useful tools for screening the population at risk of developing CVD, the literature still diverges as to which would be the best parameter to be applied for this purpose.^2^Also under discussion is the need to establish specific anthropometric parameters for each sex, as well as cutoff points that are appropriate for the different stages of life and ethnic groups.^25^

These points are of great relevance to help better understanding both the low agreement in classification and the large variability in prevalence of risk for CVD, found when comparing the methods evaluated.

Despite the fact that most cutoff points used have been set for the Brazilian population, the country has large territorial dimensions, was colonized by different peoples and at various proportions. This implies the need for studies that cover and take into consideration these diverse realities, which probably influence the presentation of the anthropometric characteristics.

Another issue is that the population evaluated in this study, with young adults, is generally represented in a smaller proportion in cohorts on risk for CVD, in which the older age groups predominate. Bearing in mind that the mean age of the population evaluated was 20.9 years, perhaps it would be necessary to adjust some cutoff points for a better evaluation of risk for CVD in this population.

In this study, it is important to point out that the objective was to evaluate the agreement in risk classification for CVD based on diverse parameters; that is, the degree to which two parameters coincide relative to the results of this classification, and not how correct this classification might be, or how close it is to some gold standard.^16^

However, we highlight the fact that in our results, depending on the parameter chosen, there may be a drastic variation in the identification of possible individuals with increased risk for CVD, which, in turn, might have a strong impact on the clinical practice, especially in the field of public health.

Additionally, the collection of a multiplicity of measures aiming to minimize the underestimation of risk may be somewhat unfeasible, both due to the great amount of time involved in this process and the strain on/discomfort for the patient.^2^

## CONCLUSION

The study demonstrated a great variability in agreement in assessment and prevalence of increased risk for cardiovascular disease, based on the anthropometric parameters evaluated, in young adults, which also seems to be influenced by sex.

These results suggest the need for caution in choosing anthropometric parameters and cutoff points to assess risk of developing cardiovascular diseases in this stage of life. Studies evaluating the Brazilian population are suggested, in order to have subsidies that help in decision-making processes, with the purpose of improving applicability of these measurements in clinical practice.
