# Supplementary material for: Agreement in cardiovascular risk rating based on anthropometric parameters
Source: Einstein (Sao Paulo). 2015 Jul-Sep;13(3):376–80. doi: 10.1590/S1679-45082015AO3349 (PMC4943782; doi:10.1590/S1679-45082015AO3349)
Supplement: Translation

# Translation: Concordância na avaliação de risco cardiovascular a partir de parâmetros antropométricos

**Authors:** Endilly Maria da Silva Dantas, Cristiane Jordânia Pinto, Rodrigo Pegado de Abreu Freitas, Anna Cecília Queiroz de Medeiros

**Affiliations:** 1 Universidade Federal do Rio Grande do Norte, Natal, RN, Brasil.

**Keywords:** Antropometria, Pesos e medidas corporais, Doenças cardiovasculares, Medição de risco, Sexo

## Abstract

**Objetivo:**

Investigar a concordância na avaliação do risco para o desenvolvimento de doenças cardiovasculares, a partir de diferentes parâmetros antropométricos, em adultos jovens.

**Métodos:**

Participaram do estudo 406 universitários, sendo aferidas as medidas de peso, estatura, e perímetros da cintura e cervical. Também foram calculados a relação cintura/estatura e o índice de conicidade. O coeficiente Kappa foi utilizado para avaliar a concordância na classificação do risco para doença cardiovascular. Também foram calculados os índices de concordância específica, positiva e negativa. O teste χ^2^ de Pearson foi utilizado para avaliar associação entre variáveis categóricas (p<0,05).

**Resultados:**

A maioria dos parâmetros avaliados (44%) apresentou concordância fraca (k=0,21-0,40) e/ou pobre (k<0,20), acompanhada de baixo valores de concordância específica negativa. A melhor concordância ocorreu entre cintura e cintura/estatura, tanto para a população em geral (k=0,88) como entre os sexos (k=0,93-0,86). Houve associação significativa (p<0,001) entre o risco para doença cardiovascular e o sexo feminino, quando utilizadas as medidas de cintura e índice de conicidade, e com o sexo masculino, quando utilizado perímetro cervical. Isso se traduziu numa grande variação na prevalência de risco para doença cardiovascular (5,5%-36,5%), a depender do parâmetro e do sexo avaliado.

**Conclusão:**

Houve variabilidade na concordância da avaliação de risco para doença cardiovascular, estabelecido a partir de parâmetros antropométricos, o que parece também ser influenciado pelo sexo. Sugere-se a condução de mais estudos, em população brasileira, para melhor compreensão desta questão.

## INTRODUÇÃO

Desde a década de 1960, as doenças cardiovasculares (DCV) representam a principal causa de morte no Brasil, e há um aumento progressivo no número de casos. Em 2013, aproximadamente 300 mil brasileiros morreram devido a DCV, e as Regiões Sudeste e Nordeste ficaram, respectivamente, o primeiro e segundo lugar.^1^

Face a esse problema, que é prevalente em todo o mundo, algumas iniciativas têm sido tomadas para desenvolver e identificar técnicas e marcadores que possam ser utilizados para avaliação do risco cardiovascular, permitindo, assim, a triagem da população, de modo a iniciar o acompanhamento o mais precocemente possível.^2,3^

Nesse sentido, a Organização Mundial da Saúde também sugere a utilização de medidas antropométricas para a vigilância dos fatores de risco das doenças crônicas, como a DCV, além de recomendar que este acompanhamento aconteça o mais precocemente possível, particularmente em razão do aumento que vem sendo detectado na prevalência de obesidade e de doenças crônicas entre pessoas mais jovens.^4,5^

Visando operacionalizar essa recomendação, vêm sendo propostos e estudados vários parâmetros antropométricos, na tentativa de melhor avaliar a obesidade central e o risco para DCV, como o perímetro da cintura (PCin),^6,4^ perímetro cervical (PCer),^7,8^ índice de conicidade (ICon)^9^ e relação cintura-estatura (RCE).^10,11^

Apesar dos resultados promissores, no entanto, a maioria dos estudos sobre a temática é realizada em população a partir da meia idade, sendo escassos os trabalhos em adultos jovens ou que avaliem a concordância e a aplicabilidade dos pontos de corte e parâmetros existentes na população mais jovem.^3,7,10,11^

## OBJETIVO

Verificar a concordância na avaliação do risco para o desenvolvimento de doenças cardiovasculares a partir de diferentes parâmetros antropométricos em adultos jovens.

## MÉTODOS

Trata-se de um estudo do tipo exploratório, quantitativo e transversal, no qual foram avaliados 406 estudantes (135 do sexo masculino e 271 do sexo feminino) da Universidade Federal do Rio Grande do Norte (UFRN).

O protocolo da pesquisa foi aprovado pelo Comitê de Ética em Pesquisa com Seres Humanos da UFRN, sob parecer 122.536 e CAAE: 06531412.4.0000.5537, e todos os voluntários assinaram o Termo de Consentimento Livre e Esclarecido.

Os critérios de inclusão para participar do estudo foram: possuir idade igual ou superior a 18 anos, estar regularmente matriculado em um curso de graduação da universidade e não apresentar limitação que impossibilitasse a coleta das medidas antropométricas. O recrutamento dos voluntários ocorreu pela divulgação do estudo nas salas de aula e em espaços de convivência da universidade.

A avaliação antropométrica foi realizada por avaliadores treinados, sendo aferidos o peso e estatura corporais, PCer e PCin. Todas as medidas foram realizadas em duplicata e foi adquirida a média. Em caso de discrepância entre os valores obtidos, uma terceira aferição foi realizada, sendo descartado o valor divergente quando do cálculo da média das aferições.

Para verificação da estatura, foi utilizado um estadiômetro (Sanny^®^, São Paulo, Brasil) perfilado em alumínio anodizado, com dispositivo de apoio em tripé e capacidade de medição de 115 a 210cm. Os voluntários foram posicionados de costas para a haste, adotando o plano de Frankfurt.^12^

A medida do peso foi realizada em balança digital portátil (Plenna^®^, São Paulo, Brasil), com capacidade para 150kg. Os universitários foram orientados a retirarem sapatos e objetos que portassem no momento.^12^

Os perímetros corporais foram mensurados utilizando fita antropométrica inelástica em fibra de vidro, com trava e extensão de 200cm. O PCin foi aferido no ponto médio, entre a crista ilíaca e a última costela, com a leitura sendo realizada no momento final da expiração.^13^ Para verificação do PCer, os participantes foram posicionados no plano de Frankfurt em pé, com os braços soltos ao longo do corpo, sendo mensurada acima da proeminência da cartilagem tireóidea.^14^

As fórmulas utilizadas para calcular o ICon e a RCE, bem como os valores de ponto de corte utilizados para a avaliação do risco para o desenvolvimento de DCV, a partir dos parâmetros antropométricos, estão descritos no quadro 1.

**Quadro 1:** Marcadores antropométricos e valores de referência para avaliação do risco cardiovascular

Marcadores antropométricos	Fórmula	Valor de referência de risco	Referência
PCin (cm)	-	≥94 para homens	ABESO^(13)^
		≥80 para mulheres	
PCer (cm)	-	≥39,6 para homens	Stabe et al.^(7)^
		≥36,1 para mulheres	
RCE	RCE=PCin(cm)/E(cm)^(12)^	≥0,52 para homens	Pitanga^(15)^
		≥0,53 para mulheres	
ICon	ICon=PCin(m)/0,109√	1,25 para homens	Pitanga^(15)^
	P(kg)/E(m)	1,18 para mulheres	

PCin: perímetro da cintura; PCer: perímetro cervical; RCE: relação da cintura estatura; E: estatura; ICon: índice de conicidade; P: peso.

A análise estatística foi realizada utilizando o programa *Statistical Package for the Social Science* (SPSS), versão 19.0. Os resultados foram expressos em média, desvio padrão, mediana, percentis e porcentuais.

A avaliação da concordância na classificação do risco cardiovascular, a partir dos diferentes parâmetros antropométricos, foi realizada pelo cálculo do coeficiente Kappa, considerado o melhor índice para esse tipo de avaliação.^16^ Para interpretação dessa medida, foram adotados os critérios propostos por Altman,^17^ que classificam o valor do coeficiente Kappa em cinco categorias, de acordo com o grau de concordância: muito boa (0,81 a 1,00); boa (0,61 a 0,80); moderada (0,41 a 0,60); fraca (0,21 a 0,40) e pobre (<0,20).

Conforme recomendado por Feinstein,^16^ para melhor contextualização do valor de Kappa, também foram calculados a concordância observada e os índices de concordância específica, positiva (CPos) e negativa (CNeg). Os índices de CPos e CNeg são utilizados para auxiliar a identificar as possíveis fontes de desacordo entre resultados obtidos com a estatística Kappa e o índice de concordância geral.^16,18^

Para investigar possíveis associações entre variáveis categóricas, foi utilizado o teste χ^2^ de Pearson. A significância estatística foi considerada quando o valor de p foi <0,05.

## RESULTADOS

A maior parte da população (66,75%) foi composta pelo sexo feminino, apresentando idade média de 21,1±3,22 anos. Na tabela 1, é apresentado o perfil antropométrico dos participantes do estudo.

**Tabela 1:** Caracterização da população por sexo

Parâmetro	Homens (n=135)		Mulheres (n=271)	
	Média±DP	Mediana (p25-p75)	Média±DP	Mediana (p25-p75)
Idade (anos)	20,8±2,85	20,00 (19,00-22,00)	21,1±3,22	20,00 (19,00-22,00)
IMC (kg/m^2^)	24,40±3,70	24,19 (21,89-26,49)	22,99±3,72	22,49 (20,51-25,01)
PCin (cm)	81,70±9,23	81,00 (75,00-87,40)	76,29±9,12	75,00 (70,00-82,00)
PCer (cm)	36,95±3,29	37,00 (35,00-39,00)	32,02±2,36	31,90 (30,50-33,00)
RCE	0,47±0,05	0,47 (0,44-0,50)	0,47±0,05	0,47 (0,43-0,51)
ICon	1,15±0,06	1,16 (1,11-1,20)	1,15±0,07	1,14 (1,10-1,19)

Os resultados foram expressos em média, desvio padrão, mediana, percentil 25 e percentil 75. DP: desvio padrão; p25: percentil 25; p75: percentil 75; PCin: perímetro da cintura; PCer: perímetro cervical; RCE: relação da cintura estatura; ICon: índice de conicidade.

Na tabela 2, são apresentados os resultados da avaliação da concordância na classificação para risco de desenvolvimento de DCV, a partir dos diferentes parâmetros antropométricos avaliados. Segundo a classificação do coeficiente Kappa, não foi encontrado nenhum caso de concordância ótima entre os parâmetros avaliados. A melhor concordância (Boa) foi entre os parâmetros Pcin e RCE, tanto para a população em geral como entre os sexos.

**Tabela 2:** Concordância da classificação para o desenvolvimento de risco para doença cardiovascular, a partir de medidas antropométricas

Parâmetros		Concordância observada	Coeficiente Kappa (IC95%)	CPos	CNeg
PCin *versus* PCer	Total	0,78	0,25 (0,14-0,35)	0,35	0,87
	Masculino	0,89	0,52 (0,32-0,72)	0,62	0,94
	Feminino	0,72	0,16 (0,07-0,26)	0,24	0,83
PCin *versus* ICon	Total	0,85	0,60 (0,51-0,69)	0,70	0,90
	Masculino	0,92	0,52 (0,28-0,76)	0,56	0,96
	Feminino	0,81	0,58 (0,48-0,68)	0,72	0,86
PCin *versus* RCE	Total	0,88	0,66 (0,57-0,75)	0,73	0,93
	Masculino	0,93	0,72 (0,56-0,88)	0,76	0,96
	Feminino	0,86	0,64 (0,54-0,74)	0,72	0,91
RCE *versus* PCer	Total	0,84	0,35 (0,23-0,48)	0,43	0,91
	Masculino	0,84	0,46 (0,26-0,66)	0,55	0,91
	Feminino	0,85	0,28 (0,14-0,43)	0,34	0,91
RCE *versus* ICon	Total	0,80	0,43 (0,33-0,53)	0,55	0,87
	Masculino	0,86	0,37 (0,16-0,58)	0,42	0,92
	Feminino	0,77	0,45 (0,34-0,55)	0,58	0,84
PCer *versus* ICon	Total	0,73	0,12 (0,02-0,21)	0,24	0,84
	Masculino	0,85	0,27 (0,05-0,49)	0,33	0,92
	Feminino	0,67	0,13 (0,05-0,21)	0,21	0,79

Geral: n=406; masculino: n=135; feminino: n=271. IC95%: intervalo de confiança de 95%; CPos: índice de concordância específica positiva; CNeg: índice de concordância específica negativa; PCin: perímetro da cintura; PCer: perímetro cervical; Icon: índice de conicidade; RCE: relação cintura/estatura.

A maioria dos parâmetros avaliados (44%) apresentou valores de coeficiente Kappa classificados como concordância fraca e/ou pobre, o que foi acompanhado de baixo valores de CNeg.

Em relação à prevalência de risco aumentado para o desenvolvimento de DCV, conforme apresentado na figura 1, foi observada uma grande variabilidade no risco, a depender do sexo e do parâmetro antropométrico utilizado na avaliação. A maior classificação de risco para a população geral e sexo masculino observada pela avaliação do ICon, já para o sexo feminino, foi por meio da medida do PCin. A maior discrepância encontrada para homens e mulheres e população geral foi entre a medida de Pcin e Pcer (25,5%).

**Figura 1:** Prevalência de risco aumentado para o desenvolvimento de doença

O resultado do teste χ^2^ revelou associação significativa entre o risco para desenvolvimento de DCV e o sexo feminino, quando utilizados os parâmetros PCin (χ^2^(1)=16,33; p<0,001) e ICon (χ^2^(1)=43,48; p<0,001); e entre o risco para desenvolvimento de DCV e sexo masculino, quando avaliado o parâmetro PCer (χ^2^(1)=12,60; p<0,001). Não foi encontrada associação entre o risco para DCV e sexo, segundo a RCE (χ^2^(1)=0,01; p>0,05).

## DISCUSSÃO

Em nosso estudo, apesar de uma alta proporção de concordância observada na classificação de risco para DCV, foi observada forte tendência a valores de coeficiente Kappa classificados como concordância fraca e/ou pobre.

Esta situação tem sido chamada de “paradoxo de Kappa”, sendo aconselhada a avaliação de outros parâmetros, como os índices de CPos e CNeg, a fim de identificar as possíveis fontes de desacordo da estatística Kappa. Dessa maneira, a partir da avaliação dos valores de CPos e CNeg, é possível visualizar a consistência entre observadores e/ou métodos, principalmente em relação a tomada de decisão em direções opostas. Ou seja, o quanto concordam na classificação de quem é positivo (apresenta a um dado fator), bem como o quanto concordam em relação a classificação de quem não o possui.^16,18^

No caso, por exemplo, da avaliação da classificação de risco para DCV a partir dos parâmetros de PCin e PCer, foi encontrada uma alta concordância, com baixos valores de coeficiente Kappa. No entanto, a avaliação dos valores de CPos e CNeg permitiu identificar que essa discrepância provavelmente se deveu aos baixos valores de CPos, indicando baixa concordância na avaliação, entre os métodos, de quem era classificado como em risco aumentado para DCV.

Esta foi uma tendência que perpassou todas as discrepâncias encontradas (baixos valores de Kappa acompanhados de elevada concordância observada), sendo observados baixos valores de CPos na comparação entre os métodos (PCin *versus* ICon; RCE *versus* PCer; RCE *versus* ICon; PCer *versus* ICon). Dessa maneira, apesar dos altos valores de concordância, quando observado o resultado da estatística Kappa e dos valores de CPos e CNeg, foi encontrada uma baixa concordância na classificação do risco para DCV entre os métodos.

Essa variabilidade na concordância da avaliação do risco para DCV parece também estar relacionada a combinação parâmetro/sexo avaliado. Houve associação entre o sexo feminino e risco para DCV quando a avaliação era feita utilizando os parâmetros de ICon (p<0,001) e PCin (p<0,001), e associação com o sexo masculino, quando avaliado o PCer (p<0,001). Esse achado foi acompanhado de uma ampla variação na prevalência do risco para DCV, estimada a partir dos diferentes métodos utilizados.

Embora esses resultados revelem uma tendência a baixa concordância na avaliação de risco para DCV entre a associação dos parâmetros avaliados, estudos brasileiros vem demonstrando a existência de associação entre essas medidas e o risco para DCV. Estudo transversal, com 968 universitários do Maranhão, encontrou correlação entre PCin e RCE e fatores de risco cardiovascular como triglicérides elevados e fumo.^19^ Outro estudo, incluindo 155 adultos entre 20 e 60 anos, residentes no Rio Grande do Sul, verificou a associação entre PCer e fatores de risco para DCV, bem como com valores aumentados de PCin.^20^ Outros trabalhos nacionais também relataram correlação positiva entre parâmetros antropométricos preditores de obesidade central e fatores relacionados ao risco aumentado para DCV, como pressão arterial elevada e aumento de lípides sanguíneos.^21-23^

Nessa perspectiva, um grande estudo prospectivo realizado nos Estados Unidos, com 49.032 homens e mulheres com idade inferior a 61 anos, encontrou que a maior quantidade de gordura corporal confere maior risco para DCV, tanto em homens quanto em mulheres, independentemente do parâmetro escolhido para avaliação.^24^

No entanto, de acordo com os resultados do presente estudo, dependendo de qual parâmetro antropométrico é utilizado para a avaliação da obesidade central, e dependendo do sexo do avaliado, podem haver grandes diferenças no resultado da avaliação de risco para DCV.

Apesar de haver consenso sobre a validade da utilização de medidas antropométricas e de seu impacto positivo enquanto ferramentas úteis para triagem populacional de risco para DCV, a literatura ainda diverge sobre qual seria o melhor parâmetro a ser utilizado para esse fim.^2^ Também vem sendo discutida a necessidade de se estabelecerem parâmetros antropométricos específicos para cada sexo, bem como de pontos de corte adequados para os diferentes estágios de vida e etnias.^25^

Estes pontos são de grande relevância para ajudar a compreender melhor tanto a baixa concordância na classificação quanto a grande variabilidade na prevalência de risco para DCV encontrados na comparação entre os métodos avaliados.

Apesar de, em sua maioria, terem sido utilizados pontos de corte estabelecidos em população brasileira, o Brasil é um país de grande dimensão territorial, que foi colonizado por diferentes povos, em diferentes proporções. Isso pode implicar a necessidade de estudos abrangendo e considerando essas diversas realidades, que, provavelmente, influenciam na apresentação das características antropométricas.

Outra questão é que a população avaliada neste estudo, de adultos jovens, geralmente está representada em menor proporção em coortes sobre risco para DCV, nas quais predominam grupos etários mais velhos. Tendo em vista que a média de idade da população avaliada foi de 20,9 anos, talvez seja necessária alguma adaptação nos pontos de corte para uma melhor avaliação do risco para DCV nesse público.

Vale salientar que, neste estudo, o objetivo foi avaliar a concordância entre a classificação de risco para DCV a partir dos diversos parâmetros, ou seja, o quanto dois parâmetros coincidem em relação aos resultados dessa classificação, e não o quão correta é essa classificação, ou o quanto se aproxima de algum padrão-ouro.^16^

Apesar disso, chama a atenção, em nossos resultados, que, dependendo do parâmetro escolhido, pode haver uma drástica variação na identificação de possíveis indivíduos com risco aumentado para DCV, o que, por sua vez, pode ter um forte impacto na prática clínica, particularmente no campo da saúde pública.

Ademais, a coleta de uma multiplicidade de medidas visando minimizar a subestimação do risco pode ser algo bastante inviável, tanto pelo grande dispêndio de tempo envolvido nesse processo como pelo desgaste/desconforto do paciente.^2^

## CONCLUSÃO

Foi encontrada uma grande variabilidade na concordância da avaliação e prevalência de risco aumentado para doença cardiovascular, a partir dos parâmetros antropométricos avaliados, em adultos jovens, o que também parece ser influenciado pelo sexo.

Esses resultados sugerem necessidade de cautela na escolha de parâmetros antropométricos e pontos de corte visando à avaliação de risco para desenvolvimento de doença cardiovasculares, nesse estágio de vida. Sugere-se a condução de estudos com esse público, em população brasileira, para que sejam construídos subsídios que auxiliem nessa tomada de decisão, no intuito de melhorar a aplicabilidade dessas medidas na prática clínica.
